# Higher Prevalence of Cognitive Impairment in Residents of High-Altitude Regions

**DOI:** 10.3390/oxygen6030016

**Published:** 2026-06-24

**Authors:** Margot Evelin Bernedo-Itusaca, Judith Marie Merma-Valero, Tatiana Milagros Cruz-Riquelme, Rocio Milagros Ccorimanya-Suni, Maria Emilia Pancaya-Flores, Zhenia Milagros Guevara-Mamani, Doris Chambi-Rodrigo, Mahely Adriana Coa-Coila, Wilma Apaza-Cansaya, Mirian Milagros Apaza-Quispe, Dante Elmer Hancco-Monrroy, Carlos Angel Loayza Coila, Alberto Salazar-Granara, Moua Yang, Ginés Viscor, Ivan Hancco Zirena

**Affiliations:** 1 Facultad de Medicina Humana, Universidad Nacional del Altiplano, Puno 21000, Peru; 2 ACEM (Asociación Científica de Estudiantes de Medicina), Universidad Nacional del Altiplano, Puno 21000, Peru; 3 Facultad de Medicina, Universidad Nacional de San Agustín, Arequipa 04000, Peru; 4 Facultad de Medicina Humana, Universidad de San Martín de Porres, Lima 15001, Peru; 5 Centro de Investigación en Medicina de Altura (CIMA), Facultad de Medicina Humana, Universidad de San Martín de Porres, Lima 15001, Peru; 6 Bloodworks Northwest Research Institute, Seattle, WA 98102, USA; 7 Division of Hematology and Oncology, Department of Medicine, University of Washington School of Medicine, Seattle, WA 98195, USA; 8 Secció de Fisiologia, Departament de Biologia Cel·lular, Fisiologia i Immunologia, Facultat de Biologia, Universitat de Barcelona, E-08028 Barcelona, Spain

**Keywords:** chronic hypoxia, cognitive impairment, excessive erythrocytosis, sleep quality, MoCA, high altitude

## Abstract

Introduction: A major health issue in individuals living at high-altitude regions is an increase in the number of red blood cells (RBCs). This condition generates a series of physiological alterations including the nervous system, where damage can occur due to increased blood viscosity. This increased viscosity, in turn, could compromise oxygen uptake, potentially linked to a degree of cognitive impairment. Objective: To determine the association between exposure to chronic hypoxia and sleep quality with the degree of cognitive impairment in a young adult population residing at different altitude levels. Methodology: A cross-sectional study was conducted with 200 apparently healthy subjects (aged 21–26 years) permanently residing in four Peruvian cities: Lima (154 m), Arequipa (2335 m), Puno (3820 m), and La Rinconada (5100 m) (*n* = 50 per location). Physiological profiles (SpO_2_, blood pressure, heart rate, hemoglobin, and hematocrit) were measured. Cognitive impairment and sleep quality were evaluated using the Montreal Cognitive Assessment (MoCA) and the Pittsburgh Sleep Quality Index (PSQI). Sex-stratified hierarchical multiple linear regression models with bootstrapping were utilized for independent correlation analysis. Results: Hemoglobin levels gradually increased with altitude, peaking at 19.47 ± 3.01 g/dL in La Rinconada, while SpO_2_ decreased to 81.64%. Moderate-to-severe cognitive impairment was exclusively restricted to the extreme altitude population of La Rinconada, where only 10% of subjects remained unaffected. In the sex-stratified multivariate regression, residency in La Rinconada initially served as a robust negative predictor of MoCA scores among women (β = −5.52, *p* < 0.001); however, this geographical effect lost statistical significance after adjusting for biological variables in Model 2 (β = −4.72, *p* = 0.178). In the fully adjusted models, neither individual hemoglobin levels nor SpO_2_ fluctuations displayed an independent linear association with cognitive performance in either sex (*p* > 0.05). Sleep quality was poor across cohorts but showed no significant association with cognitive impairment (*p* = 0.174). Conclusions: Chronic exposure to severe hypoxia (>5000 m) is associated with a greater presence of cognitive impairment, which is largely accounted for by individual physiological adaptations rather than isolated, linear effects of independent hematological or subjective sleep parameters.

## 1. Introduction

Ascent to and permanent residence in high-altitude regions expose the body to reduced barometric pressure, compromising the partial pressure of inspired oxygen and generating a state of persistent hypobaric hypoxia [1]. To counteract this deficit, the hematological system activates a compensatory response mediated by erythropoietin to increase red blood cell (RBC) mass. However, in a significant percentage of the Andean population, this response becomes maladaptive, leading to excessive erythrocytosis (EE) that alters blood rheology and increases cerebral vascular resistance [2,3].

The relationship between hyperviscosity and neurological function is complex. Although high hemoglobin (Hb) levels aim to maximize arterial oxygen content, the increased hematocrit leads to a decrease in overall cerebral blood flow. This reduction limits glucose delivery to the cortex, a crucial aspect considering the unique glucose metabolism observed in high-altitude residents. [4–6] Recent studies have shown that this microvascular hypoperfusion is directly associated with impaired memory and processing speed [7].

A key, often underestimated factor is the disruption of sleep architecture in hypoxic environments. Nocturnal hypoxia is associated with a pattern of frequent micro-arousals that fragment rest and induce periodic breathing, the role of which in sleep is still debated [8]. According to research using the Pittsburgh Sleep Quality Index (PSQI), high-altitude residents with scores indicative of poor sleep quality (PSQI > 5) exhibit exacerbated cognitive impairment, particularly in processing speed and memory consolidation. This phenomenon suggests that poor sleep quality acts as a synergistic stressor to environmental hypoxia, potentiating neuroinflammation and oxidative stress [9,10].

Despite this evidence, it remains unclear whether cognitive impairment (CI) is a linear consequence of or the result of an interaction between critical hemoglobin (Hb) thresholds, oxygen saturation (SpO_2_), and systematic sleep quality degradation. Most studies have not simultaneously integrated these variables in native populations exposed to different barometric pressure gradients.

Therefore, the present study aimed to evaluate the impact of a hypoxic environment on cognitive ability in healthy individuals residing at different altitudes. It sought to determine the association between hematological parameters, PSQI scores, and neuropsychological performance, in order to identify which factors have the greatest impact on the manifestation of cognitive impairment in the Andean population.

## 2. Materials and Methods

### – Study population:

The present research was designed as a cross-sectional observational study, conducted across four Peruvian cities situated at varying altitudes: Lima (154 m), Arequipa (2335 m), Puno (3820 m), and the town of La Rinconada (5100 m). To recruit volunteers at each study location, a convenience sampling approach was implemented, overseen by a trained local investigator. Identification of potential participants was facilitated through community outreach initiatives.

Participants were required to be apparently healthy adults of both sexes, aged 21 to 26 years, and permanently residing (defined as continuous residence of at least 12 months) in the respective study city. All participants were required to have completed more than 12 years of formal education Exclusion criteria were: self-reported history of psychiatric disorders, including depression, anxiety disorders, or traumatic brain injury; previously diagnosed sleep disorders; decompensated chronic systemic diseases; regular use of psychotropic medications or hypnotics; harmful substance use, defined as frequent alcohol consumption (>14 standard drinks/per week for men, >7 for women), active tobacco smoking, or illicit drug use; and sensory or motor limitations that would prevent adequate administration of the MoCA or PSQI instruments. Eligibility was assessed through a structured, interviewer-administered clinical data form completed by trained field personnel prior to enrollment.

### − Procedure:

Sociodemographic data, including age, sex, and length of residence in the study cities (Lima, Arequipa, Puno, and La Rinconada), were collected through direct interviews and the completion of a clinical data sheet. Anthropometric and vital sign assessments were performed following standardized protocols. Systolic and diastolic blood pressure (SBP and DBP) and heart rate (RR) were recorded using a Riester Ri-Champion adult digital upper arm sphygmomanometer (measurement range 30–280 mmHg and heart rate 40–200 bpm) after a period of rest. Oxygen saturation (SpO_2_) was measured using a Nellcor^®^ OxiMax^®^ (Boulder, CO, USA) N-65 portable pulse oximeter (Digicare Biomedical brand, 1% saturation resolution, and heart rate range 30–235 bpm). For anthropometric assessment, a Camry EB9068–59 digital scale and a fixed stadiometer were used to determine weight and height, respectively. Body mass index (BMI) was subsequently calculated from these data. For hemoglobin (Hb) level determination, a capillary puncture was performed. The area (middle or ring finger) was disinfected beforehand with an alcohol-moistened swab, and the puncture was made with a sterile lancet. The first two drops were discarded to avoid dilution with interstitial fluid, ensuring that the third drop had sufficient volume to fill the microcuvette by capillary action. The measurement was performed immediately using a HemoCue HB 201+ portable hemoglobinometer, based on the azide-methemoglobin method, with a measurement range of 0 to 25.6 g/dL. Hematocrit was measured from the same capillary blood sample using a HemataStat II microcentrifuge (EKF Diagnostics, Magdeburg, Germany); the capillary tube was filled and centrifuged according to the manufacturer’s specifications, and the packed cell volume was read using the integrated reading chart.

Neuropsychological and sleep quality assessments were performed using validated instruments administered by the patient. The MoCA was used to screen for cognitive impairment, evaluating domains such as attention, concentration, executive functions, memory, language, visuospatial skills, abstraction, calculation, and orientation. Sleep quality was assessed using the PSQI, considering its seven components to obtain an overall score.

Finally, for the classification of the participants, the cut-off criteria for EE established in the current International Consensus on Chronic Mountain Sickness [11] were considered, adjusting the reference values according to the altitude level of each city of residence.

### − Statistical analysis:

A database was created in Excel format to record the results, following the established protocol. Measures of central tendency were calculated for each variable. Analysis of variance (ANOVA) was used to compare means between more than two groups, while the chi-square test was used to establish the association between categorical variables. A multiple linear regression model, stratified by sex, was applied to evaluate the relationship between the dependent and independent variables. To ensure the robustness of the results, the regressions were performed using bootstrapping with 1000 iterations. Additionally, the Benjamini–Hochberg False Discovery Rate (FDR) correction was used to adjust *p*-values and control for multiple comparisons. Statistical processing was performed using Python (version 3.0).

### − Ethical aspects:

Prior to the start of the study, participants were fully informed about the objectives and procedures, and informed consent was obtained from each of them. The research protocol was approved on 15 April 2025 by the Ethics Committee of the University of San Martín de Porres, and received Federal Guarantees for the Protection of Human Subjects (FWA No. 00015320) and was registered with the Institutional Review Board of the U.S. Department of Health and Human Services (IRB/HHS No. 00003251).

## 3. Results

A total of 200 apparently healthy participants of both sexes were included in the study, distributed as follows: 25 women and 25 men in Lima; 22 women and 28 men in Arequipa; 34 women and 16 men in Puno; and 18 women and 32 men in La Rinconada.

Regarding anthropometric characteristics, it was observed that the age ranged between 21 and 26; subjects residing at higher altitudes exhibited a significantly higher BMI compared to residents at lower altitudes (Table 1).

**Figure 1.**
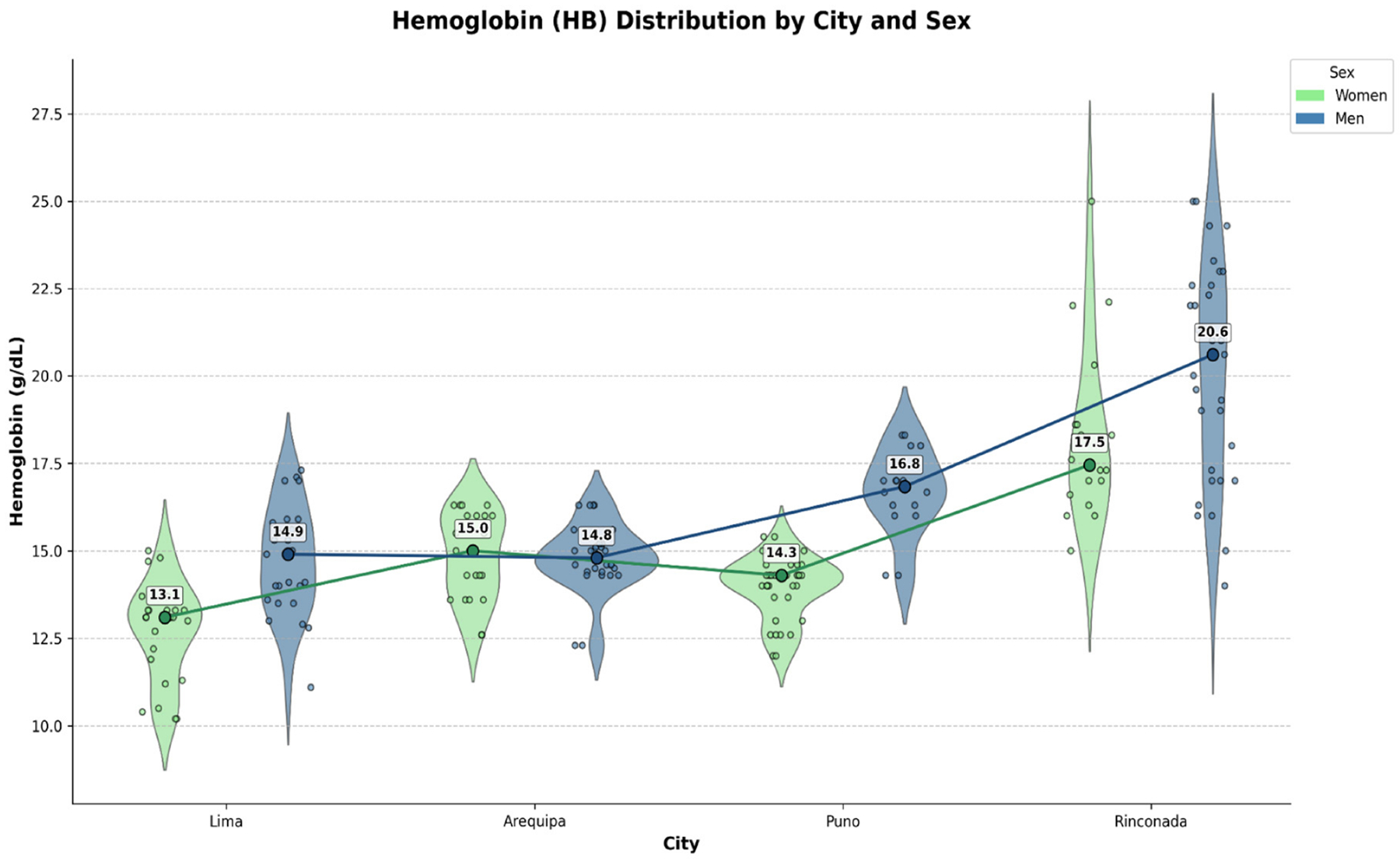
Comparison of hemoglobin levels by sex at different altitudes. Numerical labels indicate the median hemoglobin (g/dL) for each population. Trend lines connect the medians to show how physiological changes vary by geographic location.

Concerning vital signs, HR increased with altitude, with the highest values recorded in La Rinconada. Oxygen saturation (SpO_2_) decreased progressively, with the highest value in Lima (154 m) and the lowest in La Rinconada (5100 m). Simultaneously, hemoglobin (Hb) levels gradually increased, reaching a maximum mean of 19.47 ± 3.01 g/dL in the highest altitude population, demonstrating a robust erythropoietic response Puno (Figure 1). Simultaneously, hematocrit levels were significantly higher in the population of La Rinconada, reaching a mean of 58.26% ± 9.06%, which represents a marked increase compared to residents of Lima, Arequipa, and Puno (*p* < 0.001) (Table 2).

In the hemodynamic assessment, both SBP and MAP were significantly higher in the higher altitude group, while DBP showed no significant differences between sites (*p* = 0.176) (Table 2).

Regarding cognitive performance assessed via the Montreal Cognitive Assessment (MoCA), moderate-to-severe cognitive impairment was exclusively observed within the La Rinconada cohort, where only 10.0% of participants exhibited normal cognitive function. Sex-stratified analysis indicated distinct patterns of impairment: mild cognitive impairment was notably more prevalent in men (75.0%), whereas moderate cognitive impairment predominated among women (38.9%). Severe cognitive impairment was rare, with only a single case recorded in men and one case in women. Consequently, La Rinconada accounted for the highest overall burden of cognitive impairment among the studied populations (Table 3).

**Figure 2.**
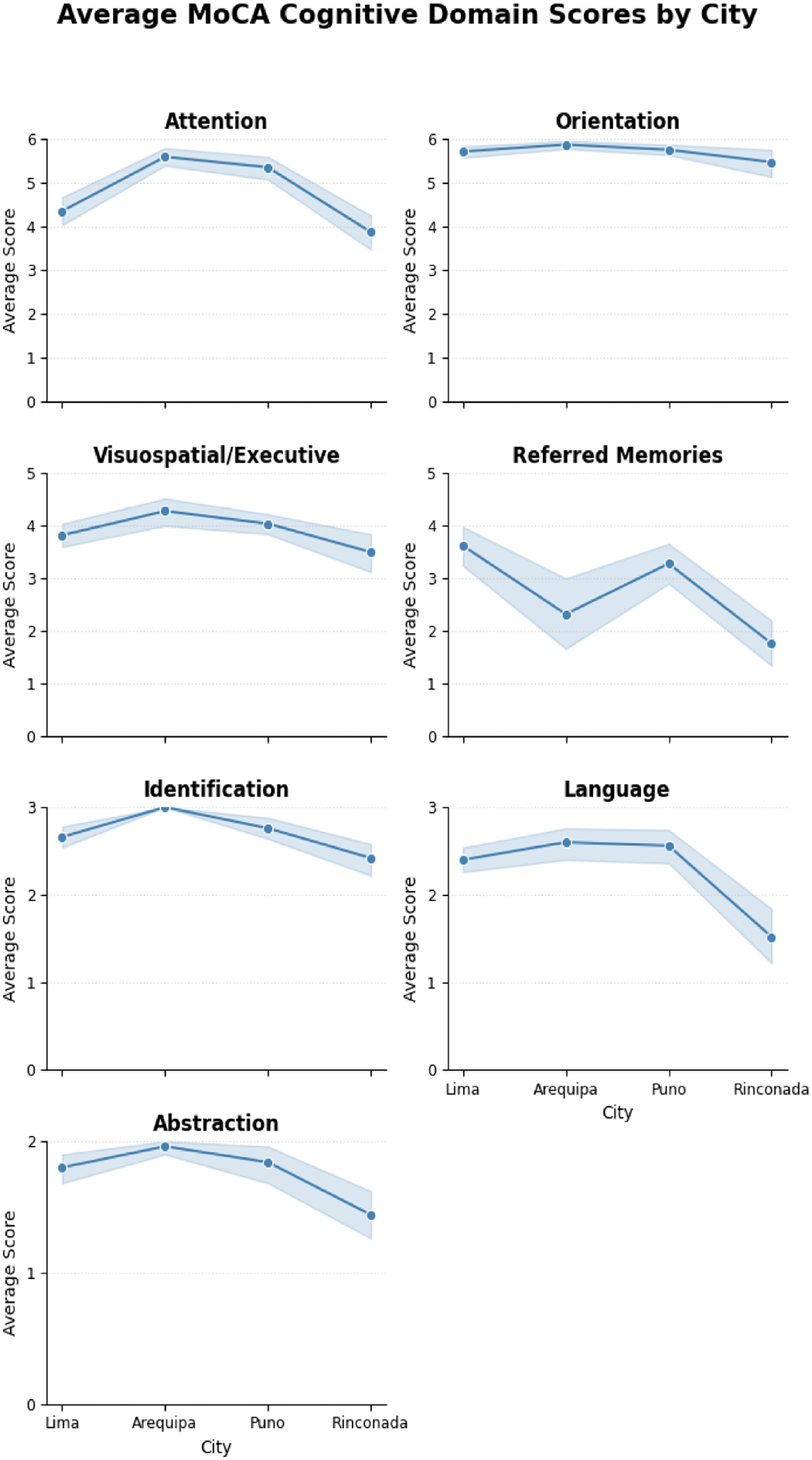
Detailed cognitive profile by MoCA domains according to city of residence. The average score obtained in seven cognitive domains is shown. The lines connect the averages across Lima, Arequipa, Puno, and La Rinconada, while the shaded areas represent the confidence interval or variability of the group.

It was observed that scores in the Visuospatial/Executive, Identification, and Attention domains were lower in La Rinconada and Lima, followed by Puno and Arequipa. In Abstraction, La Rinconada and Puno showed the greatest deficits, followed by Arequipa and Lima, while in Referred Recall and Orientation, the greatest deficits were also observed in La Rinconada and Lima, followed by Arequipa and Puno (Figure 2).

In the group without excessive erythrocytosis, mild cognitive impairment (MCI) predominated, followed by individuals without impairment, while cases of moderate and severe impairment were minimal. In the group with excessive erythrocytosis, values were low in all categories, with no statistically significant difference (Table 4).

The bar chart reflects this distribution, showing a high peak in mild cognitive impairment in the Without EE group and very low frequencies in the With EE group (Figure 2) Finally, the data suggest that, although mild cognitive impairment is more frequent in those without excessive erythrocytosis, the difference between the two groups does not reach statistical significance (Table 4).

To identify the primary predictors of global cognitive status, multiple linear regression models were run employing a bootstrapping technique (1000 iterations), with Lima (sea level) serving as the reference category. To control for Type I error inflation resulting from multiple comparisons, all *p*-values were subjected to Benjamini–Hochberg False Discovery Rate (FDR) correction. These models were systematically adjusted using a hierarchical approach and stratified by sex to enhance the robustness of the findings.

Within the female cohort, Model 1 (adjusted for age and BMI) demonstrated that residency in La Rinconada, characterized by extreme altitude, is associated with a significant reduction of 5.52 points on the MoCA compared to Lima (β = −5.52; 95% CI: −7.92 to −3.03; pFDR < 0.001). Conversely, permanent residence in Puno was linked to a superior score (β = 1.91; pFDR = 0.007). Upon the integration of physiological variables in Model 2, the effect of La Rinconada residency ceased to be statistically significant (β = −4.72; pFDR = 0.178), and neither hemoglobin nor oxygen saturation emerged as significant predictors of global cognitive performance (*p* > 0.05) (Table 5).

Predictive behavior in males showed differences compared to the female group. Residence in La Rinconada did not show statistically significant differences compared to Lima in any model after FDR adjustment (pFDR > 0.190). In Model 1, cities at intermediate altitudes showed strong positive associations: Puno (β = 3.65; pFDR < 0.001) and Arequipa (β = 3.47; pFDR < 0.001). However, when applying the full model (Model 2), the effect of Puno lost its formal statistical significance (pFDR = 0.069), with only Arequipa remaining as a robust positive predictor (β = 3.05; pFDR < 0.001). As in women, hemoglobin and oxygen saturation lacked explanatory impact in this group (pFDR > 0.500) (Table 5).

The Benjamini–Hochberg correction reduced the effect of chronological age, demonstrating sensitivity in line with the model. In women, age was a negative predictor in the baseline model (pFDR = 0.024) but lost significance in the full model (pFDR = 0.065). In contrast, in men, age showed borderline significance in Model 1 (pFDR = 0.065), which was consolidated in Model 2 (β = −0.38; pFDR = 0.044). Body Mass Index (BMI) proved not to be a confounding factor at all, maintaining pFDR values above 0.420 in all scenarios analyzed (Table 5).

Poor sleep quality was prevalent in all cities, being most common in Lima and Rinconada. Good sleep quality was infrequent, with Arequipa and Puno being the most notable. Statistically significant differences in sleep quality were found depending on the study location (Table 6).

Poor sleep quality was prevalent in both the group without EE and the group with EE. Good sleep quality was infrequent in both groups. No statistically significant differences were observed between sleep quality and the presence of EE (Table 7).

Regarding the relationship between cognitive impairment and sleep quality, mild cognitive impairment was predominant in both groups, being more frequent in those with poor sleep quality compared to those with good sleep quality. The absence of cognitive impairment was more common in the group with good sleep quality compared to the group with poor sleep quality. Moderate and severe impairment were infrequent in both groups (Table 8).

## 4. Discussion

Participants ranged in age from 21 to 26 years. Although minor age variations were observed across the study sites, as age-matching was unfeasible due to the limited size of the volunteer cohorts, cumulative evidence from prior studies suggests that these slight discrepancies are unlikely to significantly confound the outcomes of the administered assessments [12] (Table 1).

Regarding anthropometric parameters, subjects residing at 5100 m presented significantly higher BMI values compared to the other study groups (Table 1). Although previous evidence suggests that elevated BMI does not critically alter the final scores of the MoCA [13], to ensure methodological rigor, BMI was nevertheless included as an adjusting covariate in subsequent multiple linear regression models.

The higher prevalence of overweight in the La Rinconada cohort can be attributed to environmental and socioeconomic factors. Increased caloric expenditure necessitated by chronic exposure to severe cold acts as a compensatory thermoregulatory mechanism, while the relatively higher income derived from informal mining activity facilitates greater food consumption [14]. Furthermore, the underlying socioeconomic vulnerability of this population often drives the adoption of energy-dense, obesogenic dietary patterns, largely due to the prohibitive cost of nutritionally high-value foods in such isolated regions [14,15].

Regarding cardiovascular function, resting HR remained stable across Lima, Arequipa, and Puno, but was significantly elevated among La Rinconada residents (Table 2). Although chronic hypoxia typically prompts HR to return to sea-level baselines via enhanced parasympathetic activity, our findings at extreme altitude (5100 m) demonstrate a distinct physiological trajectory [16,17]. This persistent tachycardia suggests that severe hypobaric hypoxia induces sustained sympathetic nervous system hyperactivation that overrides vagal tone, a mechanism also reflected in the concurrent elevation of SBP within this cohort [18–21]. At the molecular level, this autonomic imbalance is associated with altered G-protein pathways, specifically, down-regulated Gs protein activity and increased Gi expression, which ultimately modulates adenylate cyclase and HR-regulatory ion channels [22].

**Figure 3.**
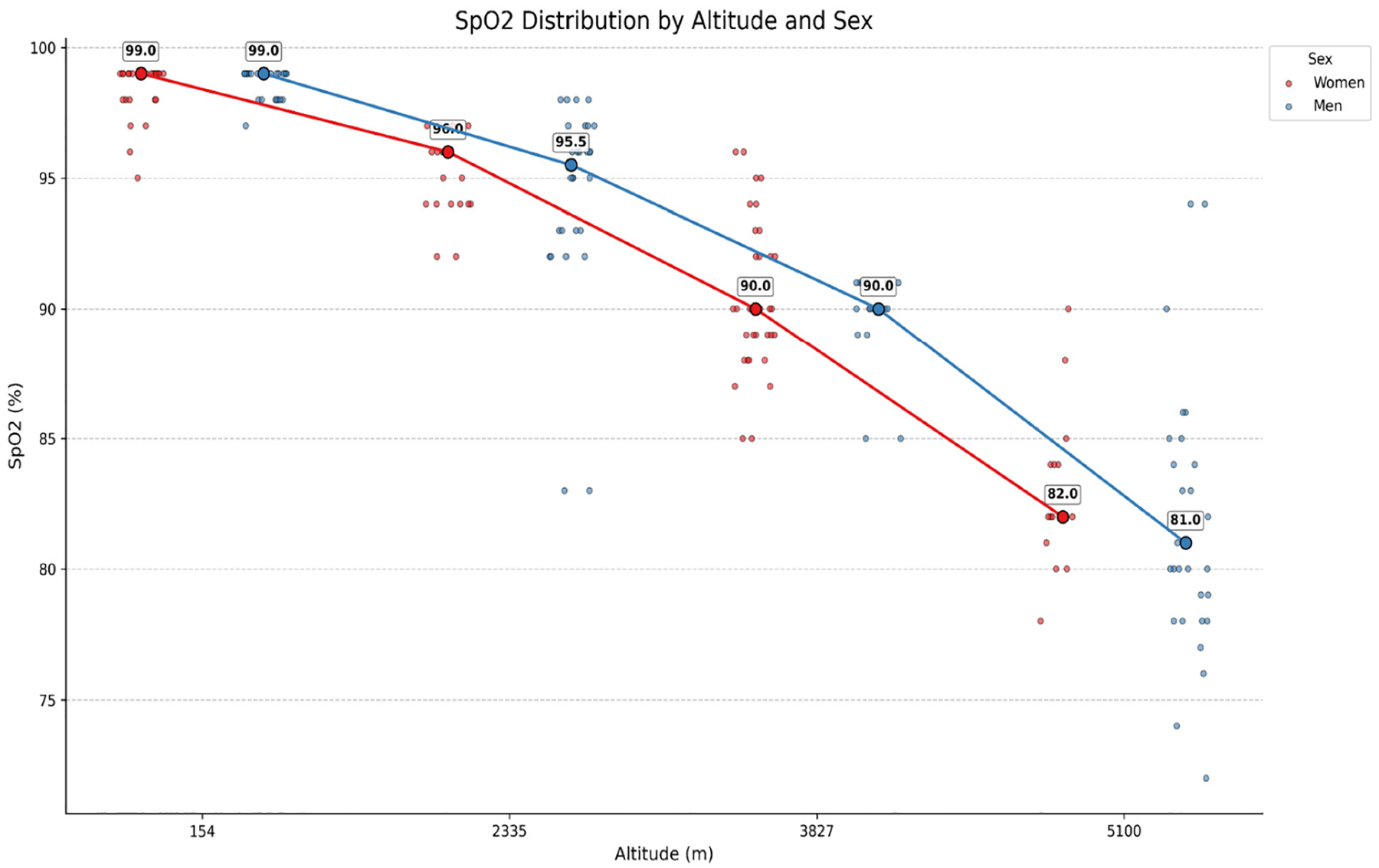
Changes in SpO_2_ in relation to altitude and sex. Numerical labels represent the median saturation for each group. Regression lines show a strong negative correlation between altitude and oxygen saturation.

**Figure 4.**
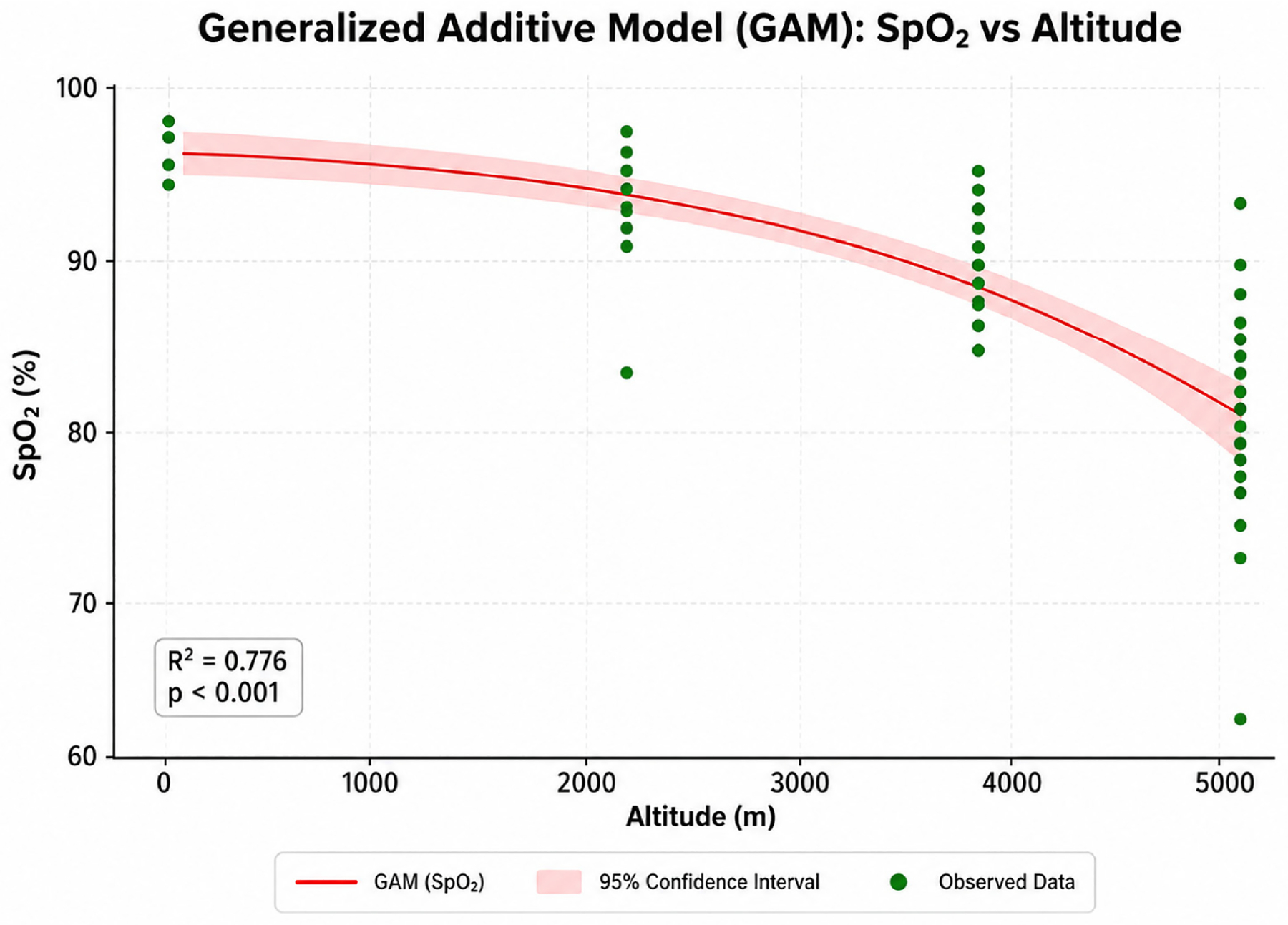
Generalized additive model (GAM) of oxygen saturation (SpO_2_) as a function of altitude. The green dots represent individual observations at the four assessed altitude levels. The solid red line indicates the trend predicted by the model, while the light red shaded area represents the 95% confidence interval (CI).

On the other hand, our results demonstrate a gradual decrease in SpO_2_, falling from 98% to 82%, a phenomenon attributable to the lower barometric pressure [23,24] (Figure 3). To determine the behavior of SpO_2_ according to altitude, a Generalized Additive Model (GAM) was applied using cubic splines (Figure 4). The model (R^2^ = 0.776, *p* < 0.001) demonstrated that the relationship between altitude and saturation is not strictly linear. It was observed that SpO_2_ decreases progressively, with the decline accelerating at higher elevations. These results allow for the establishment of more precise normative curves for different altitude populations, overcoming the limitations of previous linear models [16,17]. This physiological phenomenon corresponds to the progressive decrease in inspired oxygen pressure (PiO2) as altitude increases, with a strong linear relationship existing between hypoxia and SpO_2_ [25]. Unlike in high respiratory rate (RR), in chronic hypoxia, SpO_2_ remains persistently below baseline sea-level values, a finding consistent with previous studies [21,25]. It is important to note that at altitudes above 3000 m a.s.l., where values are significantly lower, the 90% cutoff point may be less useful.

Finally, regarding the red blood cell series, Hb concentrations progressively increased with altitude, reaching their highest mean values in La Rinconada; this finding coincides with previous reports [26,27]. Although the present study was not designed to directly evaluate molecular pathways, these findings could be interpreted within the framework of the hypoxia-inducible factor (HIF)–erythropoietin (EPO) axis, which has been proposed as one of the principal adaptive mechanisms involved in erythropoietic responses to chronic hypobaric hypoxia [28]. Furthermore, sex differences in hemoglobin levels persist at high altitude, influenced by the hormonal profile that modulates the sensitivity of the HIF-2/EPO axis [27,29].

Likewise, the elevated hematocrit observed at extreme altitude may hypothetically contribute to increased blood viscosity and altered cerebral hemodynamics. Previous studies have suggested that excessive erythrocytosis could reduce cerebral blood flow and oxygen delivery despite higher oxygen-carrying capacity [30,31]. In this context, the cognitive impairment observed in residents of La Rinconada may be associated with maladaptive hematological responses; however, causal relationships cannot be established from the present cross-sectional design.

Hemodynamic analysis revealed significant variations in systolic blood pressure (SBP) and mean arterial pressure (MAP) across the cohorts, with markedly elevated values observed in the highest-altitude population, whereas diastolic blood pressure (DBP) remained stable (Table 2). This selective increase in SBP at extreme altitude may be driven by chronic sympathetic hyperactivity, potentially triggered by chemoreceptor stimulation in response to severe hypoxia. This phenomenon could be further compounded by increased blood viscosity secondary to excessive erythrocytosis, which might elevate peripheral vascular resistance and, consequently, prompt greater ventricular ejection pressure [32,33]. Furthermore, the elevated MAP might reflect underlying arterial stiffening and endothelial dysfunction, possibly linked to reduced nitric oxide bioavailability in hypoxic environments; such alterations could theoretically predispose individuals to long-term cardiovascular events [34]. Finally, the lack of variation in DBP diverges from some existing literature describing a predominance of isolated diastolic hypertension at high altitudes [35]. This discrepancy might be partially explained by the youth of our cohort (21–26 years), who potentially retain sufficient vascular distensibility to buffer increases in diastolic pressure despite severe hypoxic stress.

**Figure 5.**
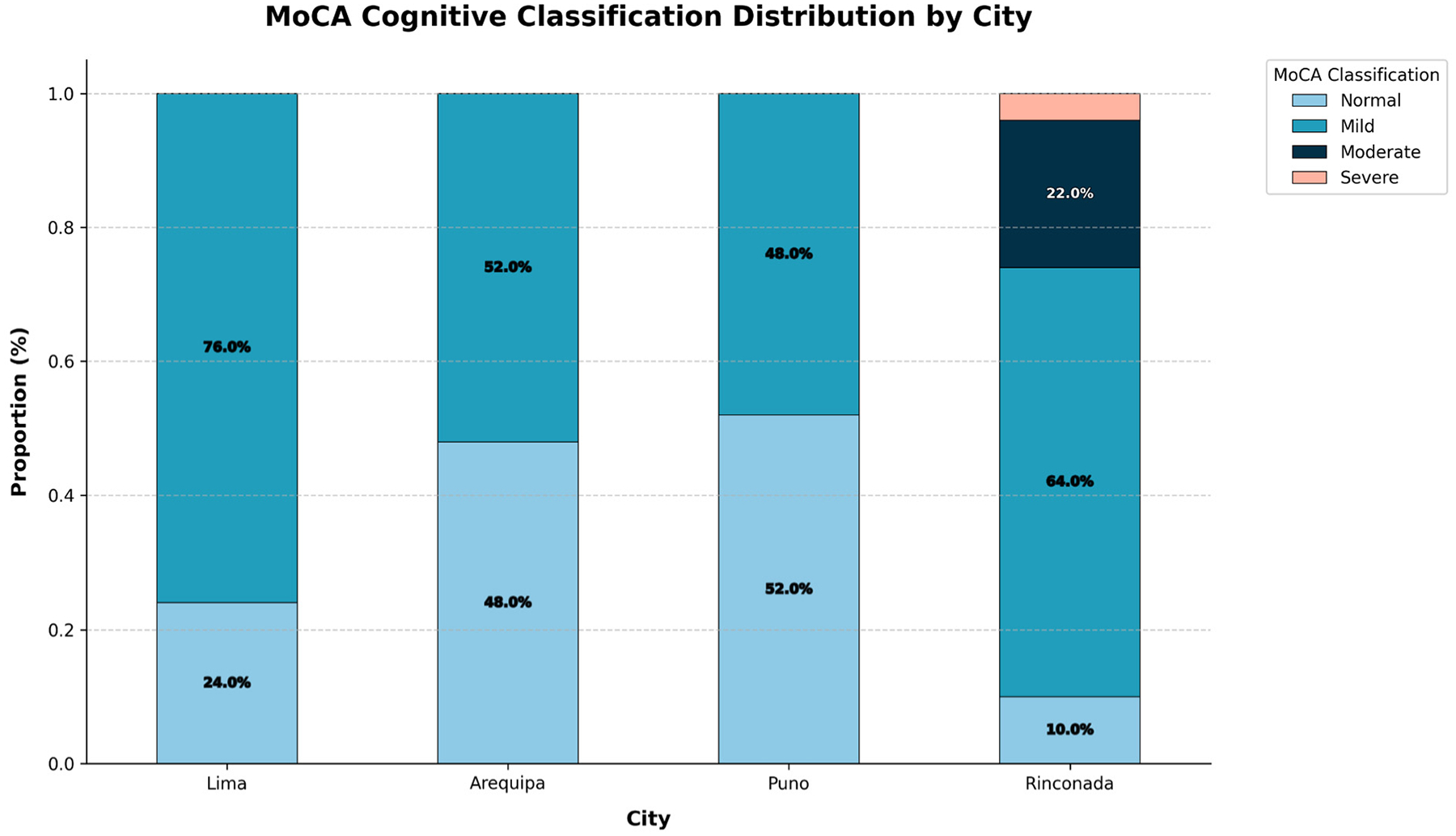
Proportional distribution of cognitive function according to the MoCA classification in the four cities with different altitude levels. The stacked bars represent the proportion (%) of subjects in the categories: Normal (light blue), Mild (medium blue), Moderate (dark blue), and Severe (light orange).

Cognitive performance assessed via the Montreal Cognitive Assessment (MoCA) exhibited statistically significant variations across the studied altitude gradients. Within the Puno cohort (3821 m), a superior proportion of subjects exhibited normal cognitive function, suggesting an effective process of functional preservation potentially linked to their status as high-altitude natives. In contrast, the population residing at the extreme altitude of La Rinconada (5100 m), comprising primarily internal migrants, demonstrated a drastic reduction in cognitively healthy subjects, who represented only 10.0% of the group. Notably, moderate-to-severe cognitive impairment emerged as a finding exclusively confined to the La Rinconada cohort (Figure 5).

These findings lend support to the hypothesis that the relationship between altitude and cognitive function may be non-linear, suggesting the potential existence of a functional threshold beyond which cerebral adaptive mechanisms might become insufficient. This contrasts with certain sensory pathways, which appear to retain a higher degree of homeostatic resilience [36]. Cumulative evidence suggests that at moderate altitudes near 3800 m, acclimatization processes may facilitate selective cognitive adaptation that attenuates global decline; conversely, chronic exposure to extreme altitudes exceeding 4000–5000 m could progressively overwhelm this adaptive capacity, potentially increasing the likelihood of neurocognitive compromise [37]. Consistent with this perspective, prior literature indicates that even moderate hypobaric hypoxia can induce early functional alterations in visual cognitive processing that are only partially compensated, further supporting the hypothesis of heightened cerebral vulnerability during more severe or prolonged exposures [38].

The unique pattern of moderate to severe impairment in La Rinconada can be pathophysiologically explained by the high prevalence of excessive erythrocytosis (Chronic Mountain Sickness) in this group (Table 3). The marked elevation of hemoglobin levels characteristic of this condition may contribute to increased blood viscosity and could hypothetically reduce cerebral perfusion and neuronal glucose delivery, a mechanism previously associated with deficits in executive function and psychomotor speed in Andean populations [30,31]. Furthermore, neuroimaging evidence suggests that severe chronic hypoxia is associated with selective gray matter atrophy within critical cortical regions, potentially accounting for the greater clinical severity observed in residents of extreme altitudes compared with lower-altitude cohorts [39].

When the results were stratified by sex according to altitude of residence and degree of cognitive impairment, distinct patterns were identified; however, due to the low sample sizes of the sex-stratified subgroups within each city, these findings must be interpreted as strictly descriptive. Statistical analysis did not reveal significant differences in the overall distribution between men and women within each city. In the sea level and Puno (3800 m) groups, the proportion of subjects without cognitive impairment was higher in women, suggesting better cognitive preservation in women at these altitudes. However, this trend was reversed at intermediate and extreme altitudes, where the proportion of cognitively healthy subjects was slightly higher in men. At 5100 m, mild cognitive impairment was markedly more prevalent in men, while moderate impairment showed the opposite trend, affecting a greater proportion of the female population. Severe cognitive impairment, although infrequent, was similarly distributed between both sexes (Table 3).

The observed sex-based variability in response to hypoxia provides preliminary descriptive insights into potential biological acclimatization mechanisms (Table 3). The trend toward superior cognitive performance among women at moderate altitudes aligns with prior literature describing heightened female neurocognitive resilience. This phenomenon is hypothesized to be linked to a robust cerebral metabolic reserve, possibly modulated by sex hormones, which might help mitigate the impact of early hypoxic stress [40]. Specifically, progesterone is recognized as a potent respiratory stimulant that enhances the hypoxic ventilatory response; this physiological pathway could theoretically improve arterial oxygen saturation and, consequently, enhance cerebral oxygenation in female cohorts [41].

Conversely, the high prevalence of mild cognitive impairment observed in men at 5100 m may be linked to their greater susceptibility to developing excessive erythrocytosis (Table 3). In this regard, androgens—particularly testosterone—are recognized to stimulate erythropoiesis, a process that, when combined with severe hypoxia, could elevate blood viscosity and compromise cerebral blood flow, potentially contributing to the greater cognitive vulnerability observed in high-altitude male residents [42].

**Figure 6.**
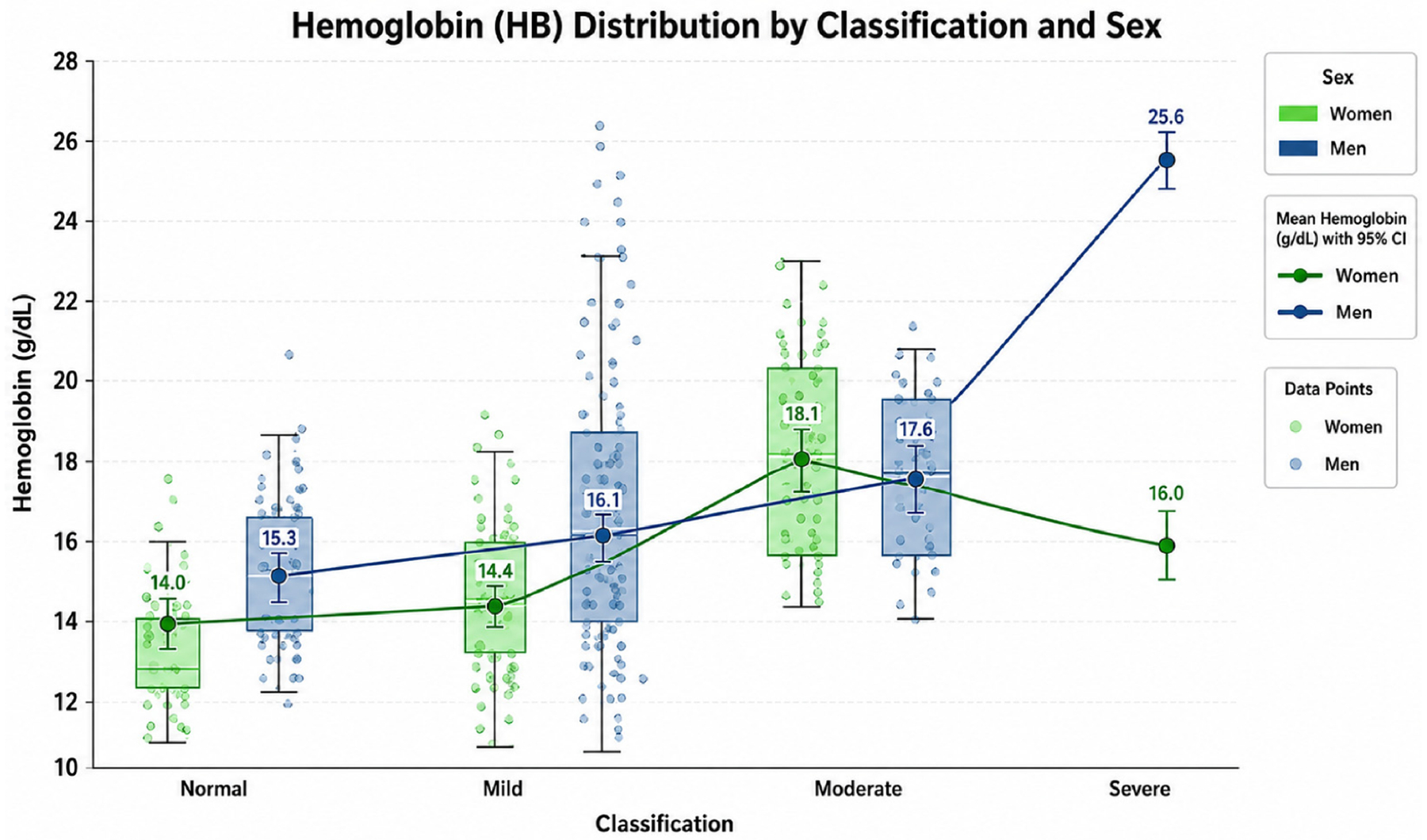
Distribution of hemoglobin levels (g/dL) according to cognitive function classification and sex (women: light green and men: blue). Numerical labels indicate the median hemoglobin for each MoCA category. Trend lines connect the medians to illustrate the relationship between the severity of cognitive impairment and hemoglobin levels.

Although classic literature posits that young women of childbearing age exhibit relative protection against both cognitive decline and excessive erythrocytosis—primarily attributed to progesterone-mediated ventilatory stimulation—our descriptive observations at the extreme altitude of La Rinconada (5100 m) suggest a divergent pattern [43,44]. In this cohort, young women exhibited a higher proportion of moderate cognitive impairment, which coincided with elevated Hb levels (18.80 g/dL) (Figure 6). Given the young age range of our participants (21–26 years), which excludes menopausal confounding, these findings theoretically point to a phenomenon of relative hemodynamic intolerance. In this scenario, an Hb concentration that might represent a manageable adaptive response in men could constitute, for young women, an extreme physiological deviation from their typical baseline (~12.0–14.0 g/dL) (Table 1). Under conditions of severe hypoxia, a potential attenuation of protective hormonal mechanisms might expose the female cerebral microvasculature to disproportionate hyperviscosity stress, thereby potentially fostering greater cognitive vulnerability; a hypothesis that aligns with recent observations in extreme-altitude populations [3].

Analysis of specific cognitive subdomains suggested a potentially non-linear relationship with altitude; residents of intermediate altitudes (Arequipa and Puno) tended to exhibit higher scores in visuospatial skills, attention, and language compared with those at sea level and extreme altitude (Figure 2). Nevertheless, the lower performance in executive function and attention observed within the La Rinconada (5100 m) cohort may point toward a potential physiological “decompensation threshold” beyond which severe chronic hypoxia might overwhelm baseline acclimatization mechanisms. While some field studies describe functional preservation driven by acclimatization, our observations suggest that such adaptive resilience might not be fully sustained under conditions of prolonged, extreme hypobaric hypoxia [45].

Crucially, although the MoCA facilitates multi-domain cognitive screening, these preliminary trends must be interpreted with strict caution. As a brief screening instrument, the MoCA is inherently limited and does not substitute for a comprehensive, gold-standard neuropsychological battery or objective neuroimaging. Consequently, these domain-specific variations, particularly in executive function, memory, attention, and visuospatial abilities, should be viewed strictly as exploratory, hypothesis-generating observations rather than definitive evidence of focal neurocognitive dysfunction.

The localized neurocognitive vulnerability observed at extreme altitude may be linked to various synergistic pathophysiological mechanisms (Figure 5). In addition to the systemic limitations imposed by diminished SpO_2_ and the elevated metabolic oxygen requirements of the brain [46], the pronounced erythrocytosis identified in the La Rinconada group could theoretically impair cerebral perfusion through hyperviscosity-mediated changes in microvascular dynamics [47,48]. Structurally, chronic exposure to severe hypoxia is associated with volumetric reductions in critical cortical regions, including the prefrontal cortex and hippocampus [49,50]. At the cellular level, such persistent hypoxic stress might trigger neuronal dysfunction and apoptotic pathways, particularly within the anoxia-vulnerable CA1 hippocampal sector, potentially driven by oxidative stress and neuroinflammation [51–53]. Collectively, these disruptions in energy homeostasis and neurotransmitter modulation could progressively overwhelm adaptive thresholds, potentially contributing to the deficits in executive function and memory consolidation observed in this population [54].

Analysis of the relationship between global cognitive performance and excessive erythrocytosis (EE) did not reach statistical significance (*p* = 0.072), although a higher prevalence of both mild and moderate cognitive impairment was observed in the EE cohort compared to subjects without this condition. Within the cohort exhibiting EE, a markedly higher prevalence of both mild and moderate cognitive impairment was identified compared to subjects without this hematological condition (Table 4). Such observations are pathophysiologically consistent with the framework of Chronic Mountain Sickness (CMS), which is not characterized merely by polycythemia but rather represents a complex multisystem syndrome. This condition encompasses varied neurocognitive manifestations, including mental confusion, impaired memory consolidation, and diminished reaction speed, stemming from persistent maladaptive responses to hypobaric hypoxia [3,55–57]. Furthermore, evidence suggests that prolonged exposure to extreme altitudes without adequate acclimatization may foster specific deficits in attention and executive processing, which are further exacerbated by significant arterial oxygen desaturation.

Analysis of mean Hb values demonstrated a statistically significant linear association, in which higher hemoglobin levels were directly linked to greater severity of cognitive impairment across both sexes (Figure 6). While prior research in general populations often describes a U-shaped association, where both anemia and polycythemia elevate the risk for cognitive decline and dementia, at high altitude, the upper end of the curve appears to be the critical determinant [58,59]. In permanent residents, excessive erythrocytosis is associated with a prothrombotic state defined by blood hyperviscosity and accelerated coagulation [60].

Excessive hemoglobin (Hb) levels have been classically associated with blood hyperviscosity, which may paradoxically reduce cerebral blood flow and oxygen delivery to neuronal tissue, potentially accelerating cognitive decline [61,62]. However, in our fully adjusted multivariate linear regression model (Model 2), individual Hb levels did not display a statistically significant independent association with MoCA scores in either women (β = 0.12, 95% CI: −0.57, 0.74) or men (β = 0.02, 95% CI: −0.43, 0.43) (Table 5). Instead, the pathophysiological relevance of the erythropoietic response is reflected in the attenuation of geographic variables. Specifically, the residence in extreme altitude (La Rinconada) was a robust negative predictor of MoCA scores among women in the demographics-adjusted model (Model 1: β = −5.46, *p* < 0.001), but this effect lost statistical significance after the inclusion of Hb and oxygen saturation (SpO_2_) in Model 2 (β = −4.65, 95% CI: −10.48, 1.21). This finding suggests that the apparent geographical impact of extreme altitude on female cognitive performance is hypothesized to be linked to individual physiological adaptations, rather than an isolated effect of hematological parameters.

Furthermore, the pattern of high-altitude cognitive vulnerability suggests a potentially divergent behavior by sex. While men exhibited a relatively stable negative trend regarding La Rinconada across both models, women demonstrated a unique susceptibility in Model 1, which was entirely accounted for once oxygen transport biomarkers were introduced. Although there is evidence of estrogen’s protective role against chronic mountain sickness through ventilatory stimulation, our results align with recent neuroimaging and functional studies indicating that women might exhibit distinct cerebrovascular reactivity thresholds and susceptibility to structural brain changes under severe chronic hypoxia [63,64]. This reinforces the clinical necessity of analyzing physiological risk thresholds through a sex-stratified approach, as hormonal, metabolic, and vascular counter-regulatory mechanisms could theoretically differ fundamentally [44].

Regarding systemic oxygenation, the previous literature indicates that chronic hypoxia is associated with compromised neurotransmitter synthesis and affects the structural integrity of key areas such as the hippocampus and prefrontal cortex [39,48]. In our stratified analysis, after controlling for residential and demographic covariates, SpO_2_ did not emerge as an independent significant predictor of MoCA scores in the final model for either women (β = 0.10, 95% CI: −0.15, 0.34) or men (β = −0.08, 95% CI: −0.34, 0.10). This may indicate that within homogenous geographic cohorts, minor individual fluctuations in saturation do not linearly dictate cognitive variance. Consequent to the observational nature of this study, causal inferences regarding independent structural alterations cannot be established. Nevertheless, the collective disappearance of the geographical significance of extreme altitude upon the introduction of SpO_2_ and Hb could theoretically underline that the complex interplay between ambient hypoxia and systemic hypoxemia might contribute to high-altitude cognitive variations [65].

Additionally, a comparative analysis of sleep quality across cities revealed a clear gradient: sleep quality worsens with increasing altitude, reaching 88.0% poor quality in La Rinconada, the highest altitude evaluated (Table 5). At high altitudes, low oxygen pressure induces instability in ventilatory control, generating what is known as “periodic breathing” or Cheyne-Stokes respiration during the night, characterized by cycles of hyperventilation followed by central apneas [66]. These apneas cause constant micro-arousals that fragment sleep and drastically reduce the deep sleep phase, resulting in a subjective perception of insufficient rest [67]. Comparative studies have shown that native highland residents have a significantly higher prevalence of central sleep apnea (77% vs. 54%) compared to lowland inhabitants, which explains the significant difference found between our lowerand higher-altitude sites [68].

Previous studies suggest that high-altitude hypoxia may contribute to periodic breathing patterns and sleep fragmentation [69].

An additional finding of interest was the significantly higher BMI observed among residents of La Rinconada compared with the other study populations. Although BMI itself was not independently associated with cognitive impairment in the present analysis, this variable may still have physiological relevance in the context of chronic hypoxia and sleep disturbances at extreme altitude. Previous evidence suggests that increased adiposity can negatively influence ventilatory efficiency, nocturnal oxygenation, and systemic inflammatory status, factors that could potentially exacerbate hypoxemia during sleep [69,70].

In residents exposed to severe hypobaric hypoxia, higher BMI may contribute to greater ventilatory instability and poorer sleep quality through mechanisms hypothesized to be linked to reduced respiratory reserve and increased susceptibility to nocturnal desaturation. Furthermore, obesity-related inflammation and endothelial dysfunction could theoretically interact with excessive erythrocytosis and elevated hematocrit, potentiating alterations in cerebral perfusion and tissue oxygen delivery.

Although no statistically significant association was observed between sleep quality and cognitive impairment in our cohort, the coexistence of elevated BMI, severe hypoxemia, and excessive erythrocytosis in La Rinconada suggests the presence of a complex multifactorial physiological environment that may contribute to neurocognitive vulnerability. Future longitudinal studies incorporating objective sleep measurements and metabolic markers are recommended to better clarify the interaction between adiposity, erythrocytosis, sleep disruption, and cognitive performance at extreme altitude.

Notwithstanding these observations, sleep quality was assessed exclusively via the PSQI questionnaire, which captures subjective sleep perception rather than objective physiological metrics. The absence of objective assessments, specifically nocturnal oximetry, polysomnography, or actigraphy, precludes the direct confirmation of sleep apnea, nocturnal hypoxemia, or sleep fragmentation. Consequently, any mechanistic interpretations regarding sleep-disordered breathing must be approached with necessary caution.

The observation that sleep quality did not differ significantly between subjects with and without EE (Table 7) aligns with the specialized literature. Prior research in CMS indicates that clinical symptom severity, including sleep disturbances, does not always correlate linearly with Hb concentrations, but is more closely linked to oxygen desaturation and global health status [71]. Specifically in La Rinconada, findings from Expedition 5300 confirm that nocturnal hypoxemia is severe and ubiquitous, affecting residents regardless of their erythrocytic status [72].

A high prevalence of poor sleep quality was observed across all cohorts, including 76.0% of cognitively healthy subjects (Table 6). This suggests that chronic hypobaric hypoxia universally disrupts sleep architecture in high-altitude populations, making poor sleep quality an environmental constant rather than a specific predictor of cognitive decline [69]. Consequently, neurocognitive vulnerability in this population is hypothesized to be linked to direct tissue hypoxemia and neuronal oxidative stress, rather than exclusively to the subjective perception of insufficient rest [58].

Limitations: Despite the robustness of the findings, several design limitations must be addressed for a nuanced interpretation. First, the cross-sectional nature of this study precludes the establishment of definitive causal trajectories between hypobaric hypoxia, maladaptive hematological responses, and cognitive performance. The use of convenience sampling introduces potential selection bias, which may constrain the ecological validity of the findings regarding the broader Andean population. A critical restriction is the lack of detailed longitudinal data on migration history, place of birth, and cumulative lifetime altitude exposure. Furthermore, other important confounding variables, such as socioeconomic status and occupational conditions, were not comprehensively assessed. The inability to fully control for these unmeasured factors limits the interpretation of the observed geographical differences. Finally, while the MoCA and PSQI allow for global screening of cognitive status and subjective sleep perception, they do not replace objective physiological parameters; specifically, this study did not assess cerebral blood flow velocity, blood viscosity, or endothelial function, nor did it utilize objective gold-standard tools for sleep measurement. Future research utilizing longitudinal designs and objective metrics, such as polysomnography, high-resolution neuroimaging, and specialized vascular assessments, is recommended to further elucidate the mechanisms identified herein.

## 5. Conclusions

This study suggests that chronic exposure to extreme altitude (>5000 m), rather than isolated hematological parameters, is strongly associated with cognitive performance in young adults. While cognitive function appears preserved at moderate altitudes (~3800 m), residence in La Rinconada is associated with a higher prevalence of moderate-to-severe cognitive impairment, a finding observed exclusively in this cohort. Adjusted multivariate regression models demonstrate that neither individual hemoglobin concentration nor oxygen saturation (SpO_2_) acts as an independent linear predictor of MoCA scores (*p* > 0.05). Instead, the erythropoietic response operates within a complex framework that may account for the observed geographical vulnerability, particularly in women. Contrary to our hypothesis, sleep quality did not show a significant association with the degree of impairment, suggesting that sleep disturbances may be a constant environmental factor at high altitude that does not independently drive cognitive variance. These results indicate a potential limit to physiological adaptation at extreme altitudes and underscore the need for comprehensive, sex-stratified systemic monitoring, highlighting that individual fluctuations in oxygenation or erythrocyte mass do not linearly correlate with lower cognitive performance in homogeneous altitudinal cohorts.

## Figures and Tables

**Table 1.** Age and body mass index (BMI) by city of residence.

	Lima	Arequipa	Puno	Rinconada	*p* Value
Age (years)	21.42 ± 1.99	23.52 ± 2.32	26.36 ± 3.31	24.94 ± 2.51	0.001 *
BMI (kg/m^2^)	24.27 ± 2.72	24.93 ± 2.79	24.48 ± 3.51	28.01 ± 5.64	0.001 *

*, statistically significant (*p* < 0.05); kg, kilograms; m, meters; BMI, body-mass-index; *p*-value, level of statistical significance.

**Table 2.** Physiological and hemodynamic parameters by city of residence.

	Lima	Arequipa	Puno	Rinconada	*p* Value
Heart Rate (bpm)	79.66 ± 12.96	77.88 ± 8.64	77.26 ± 9.38	85.40 ± 10.65	0.001 *
Oxygen Saturation (%)	98.50 ± 0.86	94.80 ± 3.05	90.20 ± 2.62	81.64 ± 5.45	0.001 *
Hemoglobin (g/dL)	13.71 ± 1.78	14.84 ± 1.08	14.82 ± 1.63	19.47 ± 3.01	0.001 *
Hematocrit (%)	41.25 ± 5.52	44.52 ± 3.24	44.46 ± 4.90	58.26 ± 9.06	0.001 *
SBP (mmHg)	110.24 ± 10.24	108.04 ± 11.76	107.44 ± 9.27	117.98 ± 17.98	0.001 *
DBP (mmHg)	76.38 ± 9.14	73.80 ± 13.76	72.40 ± 8.93	76.80 ± 13.35	0.176
MAP (mmHg)	87.67 ± 7.84	85.18 ± 11.92	84.12 ± 7.43	90.52 ± 13.95	0.015 *

*, statistically significant (*p* < 0.05); HR, heart rate; Hb, hemoglobin; Hct, hematocrit; SBP, systolic blood pressure; DBP, diastolic blood pressure; MAP, mean arterial pressure; *p*-value, level of statistical significance.

**Table 3.** Cognitive impairment classification by city, altitude, and sex.

City	Without CI	Mild CI	Moderate CI	Severe CI	*p*_a_ Value	*p*_b_ Value
LIMA (154 m)	12 (24.0%)	38 (76.0%)	0 (0.0%)	0 (0.0%)		0.000 *
Women (*n* = 25)	9 (36.0%)	16 (64.0%)	0 (0.0%)	0 (0.0%)	0.098	
Men (*n* = 25)	3 (12.0%)	22 (88.0%)	0 (0.0%)	0 (0.0%)		
AREQUIPA (2335 m)	24 (48.0%)	26 (52.0%)	0 (0.0%)	0 (0.0%)		
Women (*n* = 22)	10 (45.5%)	12 (54.5%)	0 (0.0%)	0 (0.0%)	0.973	
Men (*n* = 28)	14 (50.0%)	14 (50.0%)	0 (0.0%)	0 (0.0%)		
PUNO (3821 m)	26 (52.0%)	24 (48.0%)	0 (0.0%)	0 (0.0%)		
Women (*n* = 34)	18 (52.9%)	16 (47.1%)	0 (0.0%)	0 (0.0%)	1.000	
Men (*n* = 16)	8 (50.0%)	8 (50.0%)	0 (0.0%)	0 (0.0%)		
LA RINCONADA (5100 m)	5 (10.0%)	32 (64.0%)	11 (22.0%)	2 (4.0%)		
Women (*n* = 18)	2 (11.1%)	8 (44.4%)	7 (38.9%)	1 (5.6%)	0.137	
Men (*n* = 32)	3 (9.4%)	24 (75.0%)	4 (12.5%)	1 (3.1%)		

*, statistically significant (*p* < 0.05); CI, cognitive impairment; *p*_a_ value, level of statistical significance between men and women; *p*_b_ value, level of statistical significance between cities.

**Table 4.** Cognitive impairment classification by the presence of excessive erythrocytosis (EE).

Cognitive Impairment	Without EE	With EE	*p* Value
Without CI	64 (35.36%)	3 (15.79%)	0.072
Mild CI	107 (59.12%)	13 (68.42%)	
Moderate CI	9 (4.97%)	2 (10.53%)	
Severe CI	1 (0.55%)	1 (5.26%)	

CI, cognitive impairment; EE, excessive erythrocytosis; *p*-value, level of statistical significance.

**Table 5.** Multiple linear regression analysis with bootstrapping and Benjamini–Hochberg FDR correction for the prediction of MoCA score, stratified by sex.

Independent Variables	Women Cohort		Men Cohort	
	Model 1	Model 2	Model 1	Model 2
	β (95% CI)	β (95% CI)	β (95% CI)	β (95% CI)
Intercept	33.85 ***	21.98	29.63 ***	38.52 ***
City of Residence (Ref: Lima)				
- La Rinconada	−5.52 (−8.08, −2.98) ***	−4.72 (−10.37, 0.83)	−1.42 (−3.40, 0.76)	−2.99 (−8.08, 0.66)
- Puno	1.91 (0.66, 3.36) **	2.46 (−0.04, 4.87)	3.65 (1.48, 6.32) ***	2.86 (0.19, 5.46)
- Arequipa	0.73 (−0.79, 2.34)	0.74 (−1.10, 2.66)	3.47 (1.73, 5.28) ***	3.05 (1.26, 4.69) ***
Demographic Controls				
- Age (years)	−0.27 (−0.49, −0.07) *	−0.26 (−0.47, −0.03)	−0.39 (−0.80, −0.03)	−0.38 (−0.77, −0.04) *
- BMI	−0.12 (−0.33, 0.10)	−0.13 (−0.38, 0.12)	0.09 (−0.14, 0.31)	0.08 (−0.16, 0.28)
Physiological Variables				
- Hemoglobin (Hb)	---	0.13 (−0.50, 0.69)	---	0.01 (−0.44,0.44)
- Saturation (SpO_2_)	---	0.10 (−0.15, 0.33)	---	−0.09 (−0.38, 0.09)

β: mean bootstrap coefficient; 95% CI: 95% bootstrap confidence interval (2.5th–97.5th percentiles). *p*-Values have been adjusted using the Benjamini–Hochberg False Discovery Rate (FDR) correction. Reference level for city: Lima. Levels of statistical significance (based on p FDR):

* *p* < 0.05,

** *p* < 0.01,

*** *p* < 0.001.

BMI, Body mass index.

**Table 6.** Sleep quality classification by city of residence.

	Lima	Arequipa	Puno	Rinconada	*p* Value
Good quality sleep	0 (0.0%)	14 (28.0%)	12 (24.0%)	6 (12.0%)	0.001 *
Poor sleep quality	50 (100.0%)	36 (72.0%)	38 (76.0%)	44 (88.0%)	

*, statistically significant (*p* < 0.05); *p*-value, level of statistical significance.

**Table 7.** Sleep quality classification by the presence of excessive erythrocytosis (EE).

	Without EE	With EE	*p* Value
Poor sleep quality	152 (83.98%)	16 (84.21%)	1000
Good quality sleep	29 (16.02%)	4 (14.81%)	

EE, excessive erythrocytosis.

**Table 8.** Cognitive impairment classification by sleep quality (Pittsburgh Sleep Quality Index).

Cognitive Impairment	PSQI		*p*
	Good Quality Sleep	Poor Sleep Quality	
Without CI	16 (50.0%)	51 (30.4%)	0.1746
Mild CI	15 (46.9%)	105 (62.5%)	
Moderate CI	1 (3.1%)	10 (6.0%)	
Severe CI	0 (0.0%)	2 (1.2%)	

CI, cognitive impairment.

## Data Availability

The original contributions presented in this study are included in the article. Further inquiries can be directed to the corresponding authors.
